# Macular Vascular Geometry Changes With Sex and Age in Healthy Subjects: A Fundus Photography Study

**DOI:** 10.3389/fmed.2021.778346

**Published:** 2021-12-15

**Authors:** Ziqing Feng, Gengyuan Wang, Honghui Xia, Meng Li, Guoxia Liang, Tingting Dong, Peng Xiao, Jin Yuan

**Affiliations:** ^1^State Key Laboratory of Ophthalmology, Guangdong Provincial Key Laboratory of Ophthalmology and Visual Science, Guangdong Provincial Clinical Research Center for Ocular Diseases, Zhongshan Ophthalmic Center, Sun Yat-sen University, Guangzhou, China; ^2^Department of Ophthalmology, Zhaoqing Gaoyao People's Hospital, Zhaoqing, China

**Keywords:** macular vascular geometry, fundus photography, vascular quantification, Early Treatment Diabetic Retinopathy Study (ETDRS), normative baseline

## Abstract

**Purpose:** To characterize the sex- and age-related alterations of the macular vascular geometry in a population of healthy eyes using fundus photography.

**Methods:** A cross-sectional study was conducted with 610 eyes from 305 healthy subjects (136 men, 169 women) who underwent fundus photography examination and was divided into four age groups (G1 with age ≤ 25 years, G2 with age 26–35 years, G3 with age 36–45 years, and G4 with age ≥ 46 years). A self-developed automated retinal vasculature analysis system allowed segmentation and separate multiparametric quantification of the macular vascular network according to the Early Treatment Diabetic Retinopathy Study (ETDRS). Vessel fractal dimension (D_f_), vessel area rate (VAR), average vessel diameter (D_m_), and vessel tortuosity (τ_n_) were acquired and compared between sex and age groups.

**Results:** There was no significant difference between the mean age of male and female subjects (32.706 ± 10.372 and 33.494 ± 10.620, respectively, *p* > 0.05) and the mean age of both sexes in each age group (*p* > 0.05). The D_f_, VAR, and D_m_ of the inner ring, the D_f_ of the outer ring, and the D_f_ and VAR of the whole macula were significantly greater in men than women (*p* < 0.001, *p* < 0.001, *p* < 0.05, respectively). There was no significant change of τ_n_ between males and females (*p* > 0.05). The D_f_, VAR, and D_m_ of the whole macula, the inner and outer rings associated negatively with age (*p* < 0.001), whereas the τ_n_ showed no significant association with age (*p* > 0.05). Comparison between age groups observed that D_f_ started to decrease from G2 compared with G1 in the inner ring (*p* < 0.05) and D_f_, VAR, and D_m_ all decreased from G3 compared with the younger groups in the whole macula, inner and outer rings (*p* < 0.05).

**Conclusion:** In the healthy subjects, macular vascular geometric parameters obtained from fundus photography showed that D_f_, VAR, and D_m_ are related to sex and age while τ_n_ is not. The baseline values of the macular vascular geometry were also acquired for both sexes and all age groups.

## Introduction

The retina is one of the few tissues in the human body where the blood circulatory system can be non-invasively observed (1, 2). Retinal vascular characteristics can reflect the status of the microcirculation affected by ocular, cardiovascular, and systemic diseases, such as age-related macular degeneration, diabetic retinopathy, Alzheimer's disease, hypertension, etc. (3–11). Since early vascular alterations induced by diseases typically happened in microvasculature (12, 13), changes of the capillaries in the retinal macula are thought to be more sensitive to the early stage of disease progression and could provide significant information for the early diagnosis and monitoring of pathologic changes of related diseases (14–16).

Retinal blood vessels can be observed by imaging techniques such as fundus photography (17), optical coherence tomography angiography (OCTA) (18), fundus fluorescein angiography (FFA) (19), providing abundant retinal vascular structure and perfusion information. While OCTA and FFA could show the more detailed structure and functional changes of microvasculature, FFA is an invasive examination that may induce anaphylactic reactions (20), OCTA provides vascularity of both superficial and deep plexus but is prone to generate artifacts with a scanning imaging regime and its widespread in clinical use and disease screening is limited by the high cost, high patient cooperation and operation difficulties (18, 21). Currently, fundus photography is still the most widely used imaging technique for retinal examination and disease screening due to its advantages of non-invasiveness, relatively low cost, and ease to use the property.

The use of digital image analysis and machine learning techniques for fundus photography has become increasingly common over the past decades (22, 23), offering sophisticated techniques to acquire minute details and quantify the geometry of retinal vascular network (24, 25). Semiautomatic methods developed with Image J (26) and computer-assisted software such as Interactive Vessel Analysis (IVAN), Singapore I Vessel Assessment (SIVA), and Quantitative Analysis of Retinal Vessel Topology and Size (QUARTZ) (25, 27–29) could offer retinal vascular parameters such as vascular length, diameter, tortuosity, and so on. Clinical applications have been conducted showing these parameters may be important indicators of microvascular diseases that can be used in risk prediction (13, 15, 16). Nevertheless, most of the studies focused only on the larger blood vessels around the optic disc (5, 30).

Our former studies have developed a multiparametric retinal vascular network analysis method with a vessel segmentation algorithm based on dense block generative adversarial network (D-GAN) and automated vascular geometry quantification (14, 31). The method has been applied to explore the macular vascular geometry characteristics of fundus photography of patients with diabetes mellitus, showing microvascular morphological parameters may be indicators of the early retinal vessel changes for diabetic retinopathy (14). Though searching for disease-related potential vascular biomarkers is of great importance, the vascular geometry could be different between gender and changes could also be introduced during the normal aging process. While only a few studies have examined the potential sex and age interactions with retinal vasculatures (32, 33), no detailed research has been conducted to quantify the sex- and age-related changes of the microvascular network with fundus photography. In this study, we demonstrated a cross-sectional study aiming to explore the macular vascular geometry variations between sexes and characterized the age-related morphological changes with our multiparametric fundus photography analysis method, finding sensitive indicators of macular vascular geometry changes. Moreover, normative baseline data of both genders in different age groups was provided that can be used as references in identifying progressive macular vascular changes due to different pathologies.

## Methods

### Study Subjects

This study was approved by the Medical Ethics Committee, Zhongshan Ophthalmic Center, Sun Yat-sen University (2017KYPJ104), and adhered to the tenets of the Declaration of Helsinki. All the subjects were informed about the data collection and signed informed consent forms. A total of 610 eyes from 305 healthy patients (136 males, 169 females) were recruited from the Zhongshan Ophthalmic Center, Sun Yat-sen University and Zhaoqing Gaoyao People's Hospital, Guangdong province, China between January 1, 2019 and May 15, 2021.

The inclusion criteria were as follows: age 18–70 years old, spherical equivalent within 6.0 diopters (D), no refractive interstitial opacities that affect fundus imaging, intraocular pressure 21 mmHg or less. Ocular examinations included uncorrected and best-corrected visual acuity (BCVA) measurements, digital retinal fundus photography examination with a 50° digital fundus camera (RetiCam 3100, SYSEYE, China) without mydriasis, slit-lamp examination, and intraocular pressure measurement.

The exclusion criteria were BCVA worse than 20/25, history of ocular disease, inflammation, trauma, and any intraocular surgery. Subjects were also excluded if they had any systemic disease, such as diabetes or hypertension that could affect, or are regularly taking any medication.

The subjects were divided into four groups: Group 1 (G1) aged ≤ 25 years, Group 2 (G2) aged from 26 to 35 years, Group 3 (G3) aged from 36 to 45 years, and Group 4 (G4) aged ≥ 46 years.

### Macular Vascular Geometry Multiparametric Measurements

**Figure 1:**
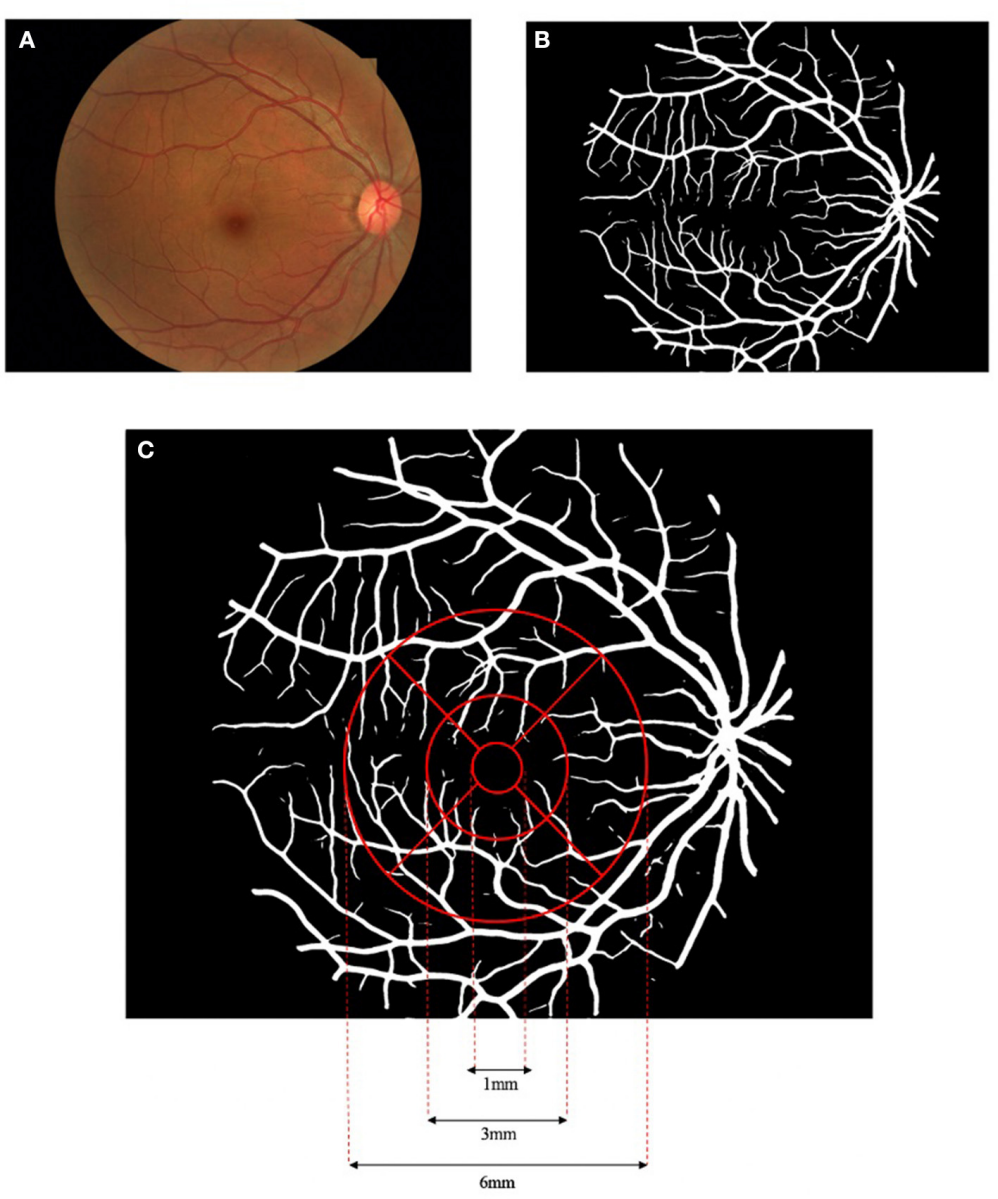
Flowchart of the macular vascular geometry assessment according to Early Treatment Diabetic Retinopathy Study (ETDRS). The fundus photograph **(A)** was pre-processed and input to the dense block generative adversarial network (D-GAN) segmentation system, exporting a binarized retinal vascular network map **(B)**, and further analyzed to quantify the macular vascular geometry based on ETDRS **(C)**.

With the color fundus photographs acquired from all the enrolled subjects, multiparametric measurements of the macular vascular geometry were performed with our self-developed fundus photography analysis software. Details of the method could refer to former publications (31). In brief, the fundus image (Figure 1A) is first processed by adaptive histogram equalization and bilateral filtering to enhance the contrast between the blood vessels and the background. The enhanced image is then input into a D-GAN-based retinal vascular segmentation algorithm, exporting a binarized retinal vascular network map (Figure 1B). The introduction of the dense blocks into the GAN enables more efficient processing between separated spatial regions of fundus images, building a more powerful generator to segment smooth, clear, and detailed blood vessels. The segmented vessel network binary image is then further analyzed by our software to quantify the macular vascular geometry based on Early Treatment Diabetic Retinopathy Study (ETDRS), separating the macular area into nine sectors (Figure 1C). The diameter of the concentric circles centered at the fovea centers are 1, 3, and 6 mm, respectively, with the inner and outer rings both evenly split into four parts (superior, inferior, nasal, and temporal). Since the innermost circle around the fovea avascular zone seldom has vessels in fundus photographs, this area is excluded for further data analysis. Vascular geometric parameters including fractal dimension (D_f_), vessel area rate (VAR), average vessel diameter (D_m_), and vessel tortuosity (τ_n_) were calculated for both the inner and outer ring as well as their sub-sectors. Note that, D_f_ is a statistic value calculated with vessel skeletons that describes the space-filling degree of a fractal, measuring the spatial availability of a complex shape, which to some extent can reflect the density and complexity of the selected microvascular network (34–36). VAR is the ratio of the vascular area to the total area of the selected region, which could comprehensively reflect vascular density while D_m_ is the mean vessel diameter of the vessels in the region. τ_n_ is defined by multiple subdivision-based algorithms calculating the maximum of the accumulated absolute tangent angle difference of the sub-segments of the blood vessels multiplied by a transformed sigmoid learning curve function of the inflection point numbers of the vessel segment curvature sign, emphasizing the human tortuosity assessment nature focusing not only global but also on local vascular features (31).

### Statistical Analysis

Statistical analysis was performed using Statistical Package for Social Sciences software (SPSS for Mac, version 26.0; IBM SPSS, Inc., Chicago, Illinois, USA). Age and sex were analyzed as classification variables while retinal vascular geometry parameters as continuous variables. Normally or approximately normal distributed variables were summarized as mean ± SD. Retinal vascular geometry parameters were assessed by the Shapiro–Wilk test.

Differences in clinical characteristics between sex at each age group were evaluated using the Independent *t-*test. The ratio of men and women in each group was analyzed by Chi-squared test. Due to the correlation nature of the included binocular data, a generalized estimating equation (GEE) procedure analysis was used with an exchangeable correlation structure to account for the inclusion of both eyes from the same participant. The final GEE model was used to calculate the β coefficients and their 95% CIs. Retinal vascular geometry parameters between the male and female subjects were evaluated through the GEE model. The association of age with D_f_, VAR, D_m_, and τ_n_ was examined using a linear GEE model. The differences in age group outcomes were analyzed, gender was included as a covariate. All the *p*-values were 2-sided and considered statistically significant when the values were <0.05.

## Results

The general age and gender characteristics of the studied subjects are shown in Table 1. There were no significant differences between the mean age of all the male and female subjects (32.706 ± 10.372 and 33.494 ± 10.620, respectively, *p* > 0.05) and the mean ages of both sexes in each age group (*p* > 0.05). Sex ratios were also equally represented in each age group.

**Table 1:** Age and gender characteristics of the studied subjects.

**Parameters**	**Male**	**Female**	***t*-statistic**	***p***
Subjects, *n* (eyes)	136 (272)	169 (338)		
Age, Mean ± SD, years	32.706 ± 10.372	33.494 ± 10.620	−0.914	0.361
**Age group, Mean** **±** **SD, years**				
Group 1 (<25)	22.896 ± 1.525 (*n* = 48)	22.778 ± 1.278 (*n* = 54)	0.601	0.548
Group 2 (26–35)	30.488 ± 2.848 (*n* = 43)	30.904 ± 2.851 (*n* = 52)	−1.000	0.318
Group 3 (36–45)	40.083 ± 3.560 (*n* = 24)	41.121 ± 2.847 (*n* = 33)	−1.669	0.099
Group 4 (>46)	51.238 ± 4.808 (*n* = 21)	52.167 ± 5.063 (*n* = 30)	−0.931	0.354

**Figure 2:**
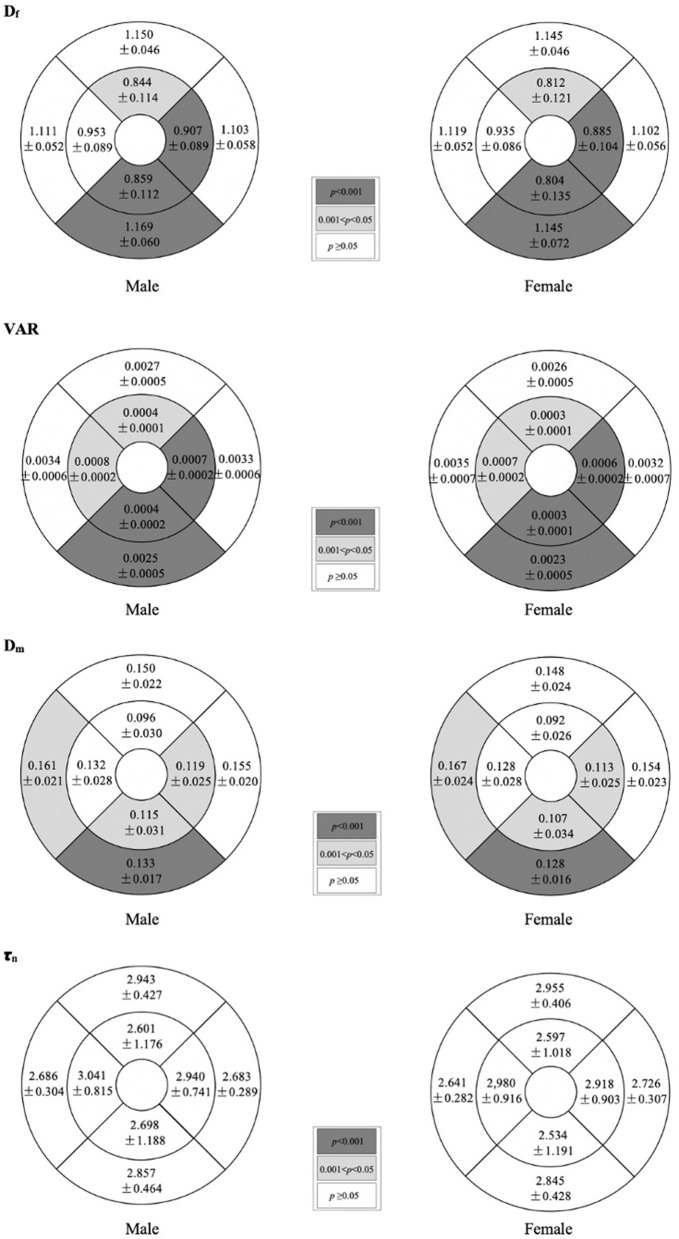
Mean ETDRS retinal vascular geometric parameters maps showing gender differences. Shaded subfields indicate those in which there was a significant difference in macular vascular geometry between the male and female (*p* < 0.05). *p*-value was based on GEE analysis accounting for the correlation between eyes of the same participant.

Table 2 shows the comparison of macular vascular geometric parameters between all the male and female subjects. Figure 2 shows the mean ETDRS retinal vascular geometric parameters maps with gender differences. The D_f_, VAR, and D_m_ of the inner ring, the D_f_ of the outer ring, and the D_f_ and VAR of the whole macular were significantly greater in males than females (Table 2; *p* < 0.001, *p* < 0.001, and *p* < 0.05, respectively), especially in the inferior quadrants (Figure 2; *p* < 0.001). There was no significant difference of τ_n_ between males and females (Table 2; Figure 2; *p* > 0.05).

**Table 2:** Generalized estimating equation analysis of the differences of macular vascular geometric parameters between the sex.

	**Males**	**Female**	**β**	**95%CI**		***p***
				**Lower**	**Upper**	
**Fractal dimension, Mean** **±** **SD, Df**
Whole	1.397 ± 0.033	1.387 ± 0.036	0.010	0.004	0.017	0.002^**^
Inner ring	1,118 ± 0.075	1.087 ± 0.085	0.030	0.015	0.045	<0.001^**^
Outer ring	1.318 ± 0.028	1.312 ± 0.031	0.008	0.002	0.013	0.009^**^
**Vessel area rate, Mean** **±** **SD, VAR**
Whole	0.0141 ± 0.0018	0.0135 ± 0.0021	5.270E−4	1.462E−4	9.081E−4	0.007^**^
Inner ring	0.0022 ± 0.0005	0.0020 ± 0.0005	2.391E−4	1.431E−4	3.364E−4	<0.001^**^
Outer ring	0.0119 ± 0.0015	0.0116 ± 0.0017	2.850E−4	−3.248E−5	6.023E−4	0.079
**Average vessel diameter, Mean** **±** **SD, Dm**
Whole	0.146 ± 0.011	0.146 ± 0.013	4.383E−4	−1.806E−3	2.683E−3	0.702
Inner ring	0.120 ± 0.015	0.115 ± 0.016	0.005	0.002	0.007	0.001^**^
Outer ring	0.150 ± 0.012	0.151 ± 0.014	−4.312E−4	−2.850E−3	1.987E−3	0.727
**Tortuosity, Mean** **±** **SD**, **τn**
Whole	2.742 ± 0.147	2.765 ± 0.152	−0.023	−0.051	0.005	0.101
Inner ring	2.945 ± 0.357	2.938 ± 0.359	0.007	−0.052	0.066	0.825
Outer ring	2.688 ± 0.158	2.707 ± 0.171	−0.019	−0.048	0.009	0.189

*p-value was based on GEE analysis using an exchangeable correlation structure for the difference between means, accounting for the correlation between eyes of the same participant*.

*CI, 95% Walder confidence interval*;

** *p <0.01, bold*.

**Figure 3:**
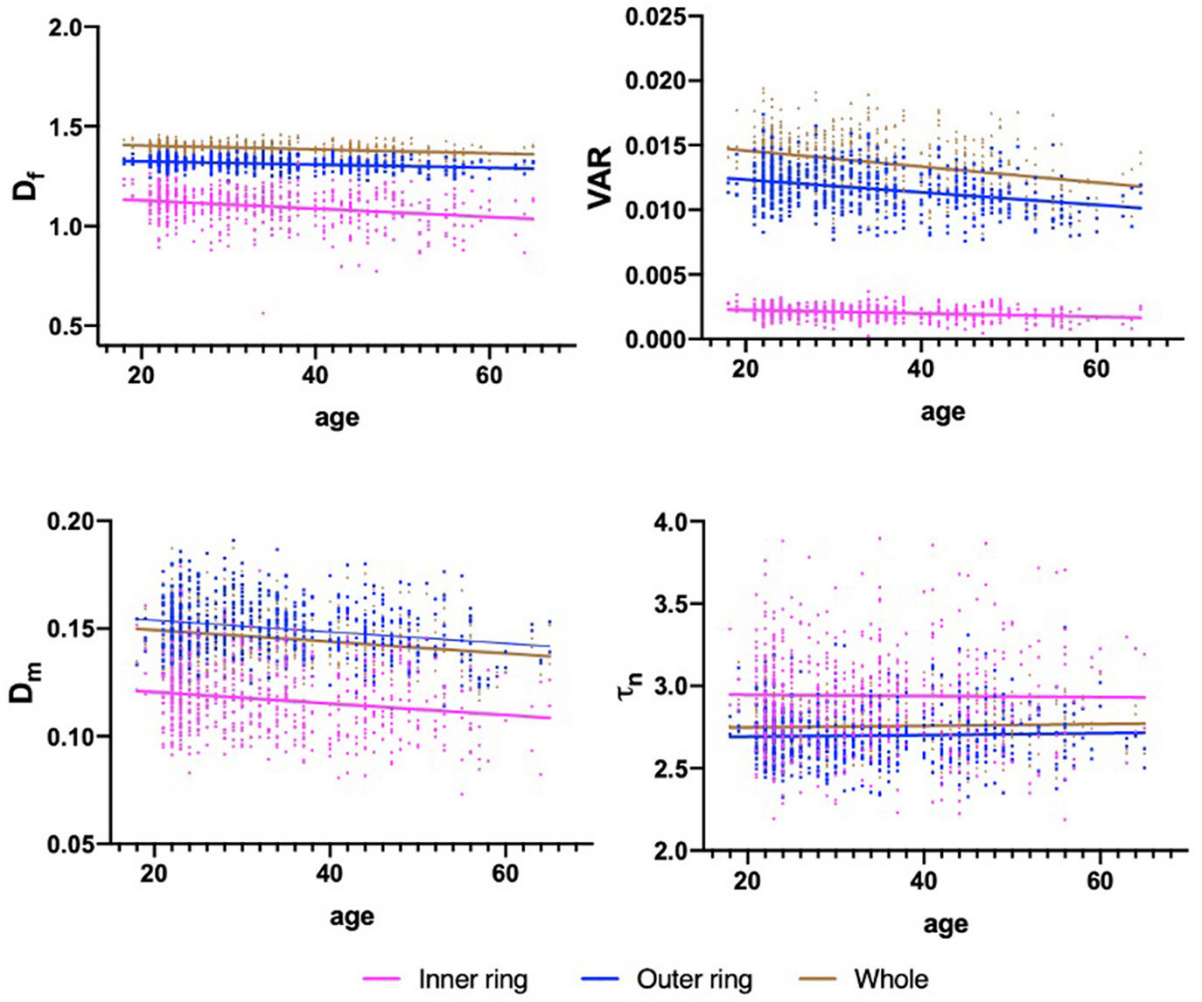
Scatterplots for the associations between macular vascular parameters and age. The significant negative association exists between the D_f_, VAR, D_m_, and age in the whole macula, inner and outer rings (*p* < 0.001), while τ_n_ showed no significant association with age (*p* > 0.05).

Table 3 shows the association results between macular vascular geometric parameters and age. The D_f_, VAR, and D_m_ of the whole macular, inner and outer rings all negatively associated with age (Table 3; Figure 3; *p* < 0.001), whereas the τ_n_ of the macular did not correlate with age (Table 3; Figure 3; *p* > 0.05).

**Table 3:** Generalized estimating equation analysis of the association between macular vascular geometric parameters and age.

	**Age**			
	**β**	**CI**		***p***
		**Lower**	**Upper**	
**Fractal dimension, D**_**f**_				
Whole	−9.812E−4	−1.276E−3	−6.873E−4	<0.001^**^
Inner ring	−2.069E−3	−2.781E−3	−1.357E−3	<0.001^**^
Outer ring	−7.904E−4	−1.043E−3	−5.372E−4	<0.001^**^
**Vessel area rate, VAR**				
Whole	−6.179E−5	−7.804E−5	−4.555E−5	<0.001^**^
Inner ring	−1.299E−5	−1.772E−5	−8.264E−5	<0.001^**^
Outer ring	−4.873E−5	−6.195E−5	−3.551E−5	<0.001^**^
**Average vessel diameter, D**_**m**_		
Whole	−2.710E−4	−3.741E−4	−1.693E−4	<0.001^**^
Inner ring	−2.652E−4	−3.861E−4	−1.440E−4	<0.001^**^
Outer ring	−2.684E−4	−3.812E−4	−1.562E−4	<0.001^**^
**Tortuosity, τ_n_**				
Whole	7.202E−4	−6.023E−4	2.042E−3	0.286
Inner ring	3.764E−4	−2.734E−3	1.981E−3	0.754
Outer ring	5.203E−4	−7.752E−4	1.815E−3	0.432

*p-value was based on GEE analysis using an exchangeable correlation structure for the difference between means, accounting for the correlation between eyes of the same participant*.

*CI, 95% Walder confidence interval*;

** *p <0.01, bold*.

**Figure 4:**
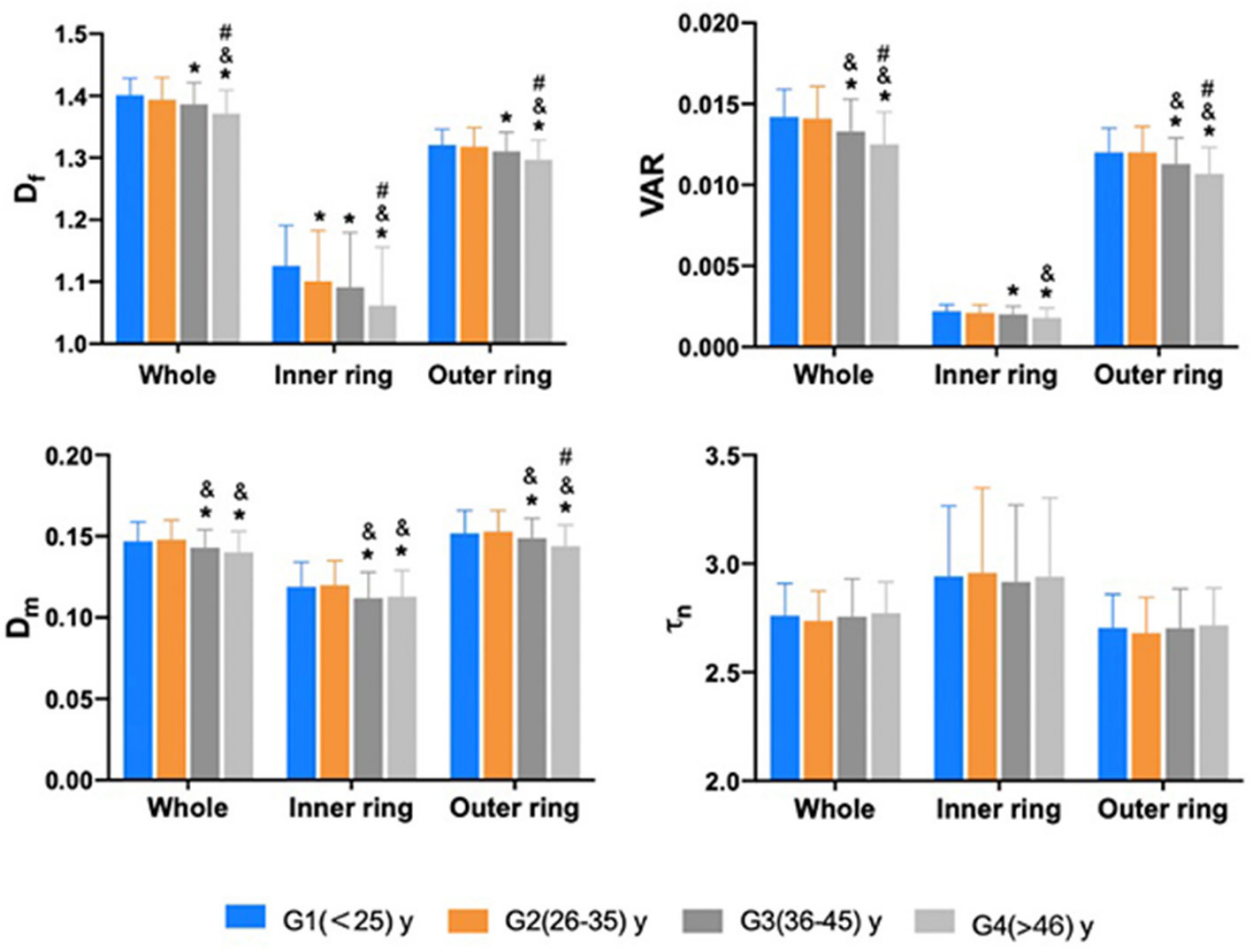
The comparison of macular vascular geometric parameters of the whole, the inner and outer rings among different age groups. Df of the inner ring showed a significant decrease in G2 compared to G1 (*p* < 0.05). D_f_, VAR, and D_m_ all decreased from G3 compared to younger groups in the whole, inner and outer rings (*p* < 0.05). τn has no significant differences (*p* > 0.05). *Compare to G1 *p* < 0.05; ^&^ compare to G2 *p* < 0.05; ^#^: compare to G3 *p* < 0.05. *p* value was based on GEE analysis accounting for the correlation between eyes of the same participant and including gender as a covariate.

The comparison of macular vascular geometric parameters of the whole, the inner and the outer rings among the different age groups are shown in Figure 4. It is resolved that, in the inner ring, G2 showed a significant decrease of the D_f_ compared with G1 (Figure 4; *p* < 0.05), while D_f_, VAR, and D_m_ all decreased from G3 compared with the younger groups in the whole, inner and outer rings (Figure 4; *p* < 0.05). τn also has no significant differences (Figure 4; *p* > 0.05).

Table 4 shows the baseline values of D_f_, VAR, D_m_, and τ_n_ of both sexes in 4 age groups.

**Table 4:** The baseline values of macular vascular geometric parameters of both the sexes in the four age groups.

**Age groups**	**Group 1**		**Group 2**		**Group 3**		**Group 4**	
	**Male**	**Female**	**Male**	**Female**	**Male**	**Female**	**Male**	**Female**
**Fractal dimension, Mean** **±** **SD, D**_**f**_								
Whole	1.400 ± 0.027	1.401 ± 0.027	1.399 ± 0.032	1.390 ± 0.038	1.398 ± 0.036	1.378 ± 0.032	1.382 ± 0.038	1.363 ± 0.037
Inner ring	1.128 ± 0.068	1.124 ± 0.061	1.116 ± 0.074	1.088 ± 0.086	1.127 ± 0.081	1.065 ± 0.083	1.090 ± 0.080	1.043 ± 0.099
Outer ring	1.320 ± 0.024	1.322 ± 0.027	1.321 ± 0.027	1.315 ± 0.033	1.318 ± 0.032	1.304 ± 0.029	1.307 ± 0.033	1.290 ± 0.030
**Vessel area rate, Mean** **±** **SD, VAR**								
Whole	0.0142 ± 0.0016	0.0143 ± 0.0018	0.0144 ± 0.0018	0.0139 ± 0.0020	0.0140 ± 0.0019	0.0129 ± 0.0019	0.0133 ± 0.0021	0.0120 ± 0.0018
Inner ring	0.0023 ± 0.0005	0.0022 ± 0.0004	0.0022 ± 0.0005	0.0020 ± 0.0005	0.0022 ± 0.0005	0.0018 ± 0.0005	0.0021 ± 0.0005	0.0017 ± 0.0006
Outer ring	0.0119 ± 0.0014	0.0121 ± 0.0016	0.0122 ± 0.0015	0.0119 ± 0.0017	0.0117 ± 0.0015	0.0110 ± 0.0016	0.0112 ± 0.0017	0.0103 ± 0.0014
**Average vessel diameter, Mean** **±** **SD, D**_**m**_								
Whole	0.145 ± 0.011	0.149 ± 0.013	0.149 ± 0.011	0.148 ± 0.012	0.145 ± 0.011	0.142 ± 0.011	0.142 ± 0.011	0.139 ± 0.013
Inner ring	0.121 ± 0.016	0.117 ± 0.014	0.123 ± 0.014	0.117 ± 0.016	0.117 ± 0.014	0.109 ± 0.016	0.115 ± 0.014	0.111 ± 0.017
Outer ring	0.149 ± 0.012	0.154 ± 0.015	0.153 ± 0.012	0.152 ± 0.013	0.151 ± 0.0119	0.147 ± 0.0126	0.146 ± 0.012	0.143 ± 0.014
**Tortuosity, Mean ± SD, τ_n_**								
Whole	2.757 ± 0.144	2.765 ± 0.151	2.729 ± 0.143	2.745 ± 0.134	2.737 ± 0.164	2.769 ± 0.183	2.743 ± 0.141	2.794 ± 0.142
Inner ring	2.921 ± 0.300	2.961 ± 0.343	2.990 ± 0.468	2.930 ± 0.308	2.910 ± 0.291	2.918 ± 0.397	2.945 ± 0.257	2.938 ± 0.425
Outer ring	2.709 ± 0.148	2.701 ± 0.161	2.662 ± 0.158	2.697 ± 0.167	2.694 ± 0.183	2.710 ± 0.180	2.691 ± 0.149	2.735 ± 0.184

## Discussion

This is the first study that quantifies the sex- and age-related macular vascular geometry alterations in healthy populations with fundus photography using our customized automatic multiparametric vascular analysis software. We demonstrated that macular vascular D_f_, VAR, and average vessel diameter D_m_ were all significantly lower in the female than male and all decrease with the increase of age, while vessel tortuosity τ_n_ showed no statistical difference, revealing the physiological variations of macular vascular geometry induced by sex and age. Normative baseline data of macular vascular geometric parameters for both the genders in different age groups were acquired that can potentially be used as references in identifying progressive macular vascular changes due to different pathologies.

In the analysis of the macular vascular geometric difference between male and female healthy subjects, we found that D_f_ and VAR in the macular area and D_m_ in the inner area of the macula were significantly greater in men than in women, indicating that male has more complex retinal vascular structures and larger vessel occupation and diameter in the macular area as compared with female. While no studies have demonstrated macular vascular structure analysis with fundus photography between genders, our results showing more abundant vascular network in men are in consistent with the former studies. Using OCTA, Yu et al. (37) reported lower blood flow index in the macula and larger fovea avascular zone in women, and Wang et al. (38) revealed higher superficial retinal vascular density in men than in women. Using fundus photography, Tapp et al. (39) showed that the retinal vascular diameter around the optic disk was larger in male. Since the retina is a tissue with high-oxygen consumption that need to rely on blood perfusion to maintain its metabolic levels, the retinal vascular structure is mainly influenced by the blood perfusion level (37, 40), which is regulated by blood pressure, intraocular pressure (IOP) (41), body mass index (BMI) (42), and hormone (43). Thus, the proven differences in these factors between genders (44) might be the reasons leading to the macular vascular geometry divergence. There were studies exploring gender differences in the epidemiology of ophthalmic diseases that have shown that macular diseases such as age-related macular degeneration (AMD) and macular holes (45, 46) were more likely to happen in females, which could also potentially be related to the macular vascular geometry differences. The detailed mechanisms of the gender-specific differences of macular vascular geometry remain unclear and require further study.

We have described the association between age and macular vascular geometric parameters showing that D_f_, VAR, and D_m_ of the macular area all decrease with the increase of age. The association between age and D_f_, VAR, and D_m_ were significant while the correlation coefficient is relatively low, indicating small age-related macular vascular geometry changes. This is similar to those reported in other studies (47, 48). One possible reason is that the age range of the samples in our study is not wide enough with the fewer elderly samples. The other reason could be that many factors influence macular vessels such as gender, axial length, refractive error (47, 49, 50), while this study limited in exploring only the age-induced changes. In the comparison of macular vascular geometric parameters of different age groups, we have revealed that statistical variation of vascular D_f_ happened from 26 to 35 age group while VAR and D_m_ decrease from 36 to 45 age group. These are in accordance with studies demonstrated in exploring retinal vascular alterations with aging (34, 51). Using fundus photography, Azemin et al. (51) and Liew et al. (34) measured the whole retinal and observed a significant decrease in the D_f_ with aging. Sun et al. (52) found both arterial and venular caliber decrease in the older groups using IVAN software to analyze peripapillary diameter of 3,019 health participants; Pose-Reino et al. (53) analyzed the retinal blood vessel caliber and found that the reduction of retinal arteriole caliber occurs during aging. While the retinal vascular structure is closely related to blood perfusion, measuring by OCTA, Yu et al. (37) have shown that macular perfusion decreased with increasing age in healthy Chinese eyes, and the parafoveal flow index and vessel area density decrease with aging at a rate of 0.6 and 0.4% per year. You et al. also reported that higher superficial and deep capillary densities were significantly associated with younger age and a general negative association between macular vessel density and age (47). Similar results have been reported in several other studies (48). Similar to other human organs, the physiological function of the eye decreases with aging. Since maintaining normal function of the retina depends on normal metabolism level (54), aging causes tissue loss and a corresponding reduction in oxygen demand, altering vascular system, and declining the perfusion of macular, resulting in a reduction in vascular structure and complexity. Studies have also determined that aging could cause thickening of the vascular wall, reducing vascular elasticity (55), and the rigidity of the vessel wall would lead to the decrease of the vessel diameter (56). The reduction in the macular vascular structure might potentially account for some ocular diseases related to age such as age-related macular degeneration (AMD), proliferative diabetic retinopathy (PDR), retinal vein occlusion (RVO), and so on (57, 58).

In this study, we also found that the macular vascular tortuosity τ_n_ has no variation between gender and across aging. Many studies have shown that retinal vascular tortuosity variation is one of the earliest indicators of a number of relevant diseases such as diabetes, cerebrovascular disease, stroke, ischemic heart disease (14, 59, 60). Our observation indicates no statistical difference of macular vascular tortuosity in healthy individuals of different gender and age further enhancing the feasibility of using tortuosity as a sensitive indicator for the early diagnosis of related diseases.

There are several limitations of this study. First, the number of subjects in the elderly age group was relatively small due to the fact that the healthy subjects we have collected tend to be young since older people are more likely to suffer from systemic diseases such as hypertension, heart disease, and diabetes or cloudy refractive interstitium affecting the fundus image quality (6, 9, 14, 34, 50). Future collection of more healthy elderly subjects to balance the sample size of all the age groups would be beneficial. Second, while this study is limited in exploring macular vascular geometry changes with age and gender, there are many other factors that affect the macular vasculature, such as axial length, refractive error, IOP, blood pressure, and so on (34, 41, 49, 50, 61), it is worth further in-depth study considering these factors. Third, since this is a cross-sectional study without temporal information, future prospective investigation with systematic clinical information acquired could provide more detailed support of the vascular alteration mechanisms. Moreover, with the binocular data acquired in this study, analyzing the intereye correlation and difference of the macular vasculature with fundus photography and comparing it to those acquired with OCTA would be interesting (62). Finally, with the baseline data from the healthy subjects gained, the vascular geometric variation induced by different diseases could be further explored. Improvement of our current vascular analysis software to distinguish the retinal arteries and veins would also provide more functional information to study the retinal vascular circulation.

In conclusion, findings from this study reveal the sex- and age-related macular vascular geometry alterations in healthy human subjects and provide the normative baseline data for both genders in different age groups. The application of our automatic retinal vascular analysis system could provide a useful approach for detecting subtle macular vessels change with fundus photography.

## Data Availability Statement

The original contributions presented in the study are included in the article/supplementary material, further inquiries can be directed to the corresponding authors.

## Ethics Statement

The studies involving human participants were reviewed and approved by Guangzhou, China 2017KYPJ104, Medical Ethics Committee of Zhongshan Ophthalmic Center, Sun Yat-sen University (Guangzhou, China). Written informed consent to participate in this study was provided by the participants' legal guardian/next of kin.

## Author Contributions

JY and PX conceived and planned the research and reviewed and modified the manuscript. ZF, GW, and HX carried out the studies and wrote the first draft of the manuscript. GL and TD conducted the fundus imaging. GW, PX, and ML processed the images and organized the database. ZF and GL performed the statistical analysis. All the authors provided critical feedback and helped to shape the research, analysis, and manuscript.

## Funding

This study was supported by the Key-Area Research and Development Program of Guangdong Province (No. 2019B010152001), the National Natural Science Foundation of China (81901788), and the Guangzhou Science and Technology Program (202002030412).

## Conflict of Interest

The authors declare that the research was conducted in the absence of any commercial or financial relationships that could be construed as a potential conflict of interest.

## Publisher's Note

All claims expressed in this article are solely those of the authors and do not necessarily represent those of their affiliated organizations, or those of the publisher, the editors and the reviewers. Any product that may be evaluated in this article, or claim that may be made by its manufacturer, is not guaranteed or endorsed by the publisher.
